# Prenatal Maternal Stress From a Natural Disaster and Hippocampal Volumes: Gene-by-Environment Interactions in Young Adolescents From Project Ice Storm

**DOI:** 10.3389/fnbeh.2021.706660

**Published:** 2021-09-10

**Authors:** Lei Cao-Lei, Sandra Yogendran, Romane Dufoix, Guillaume Elgbeili, David P. Laplante, Suzanne King

**Affiliations:** ^1^Department of Psychiatry, McGill University, Montreal, QC, Canada; ^2^Douglas Hospital Research Centre, Montreal, QC, Canada; ^3^Centre for Child Development and Mental Health, Lady Davis Institute, Jewish General Hospital, Montreal, QC, Canada

**Keywords:** prenatal maternal stress (PNMS), hippocampal volumes, COMT, BDNF, gene-by-environment interactions, natural disaster

## Abstract

Gene-by-environment interactions influence brain development from conception to adulthood. In particular, the prenatal period is a window of vulnerability for the interplay between environmental and genetic factors to influence brain development. Rodent and human research demonstrates that prenatal maternal stress (PNMS) alters hippocampal volumes. Although PNMS affects hippocampal size on average, similar degrees of PNMS lead to different effects in different individuals. This differential susceptibility to the effects of PNMS may be due to genetic variants. Hence, we investigated the role of genetic variants of two SNPs that are candidates to moderate the effects of PNMS on hippocampal volume: COMT (rs4680) and BDNF (rs6265). To investigate this, we assessed 53 children who were *in utero* during the January 1998 Quebec ice storm. In June 1998 their mothers responded to questionnaires about their objective, cognitive, and subjective levels of stress from the ice storm. When children were 11 1/2 years old, T1-weighted structural magnetic resonance imaging (MRI) scans were obtained using a 3T scanner and analyzed to determine hippocampal volumes. We collected and genotyped the children’s saliva DNA. Moderation analyses were conducted to determine whether either or both of the SNPs moderate the effect of PNMS on hippocampal volumes. We found that objective hardship was associated with right hippocampal volume in girls, and that the BDNF and COMT genotypes were associated with left hippocampal volume in boys and girls. In addition, SNPs located on COMT moderated the effect of maternal objective distress in boys, and subjective distress in girls, on both right hippocampal volume. Thus, we conclude that an individual’s genotype alters their susceptibility to the effects of PNMS.

## Introduction

The hippocampus plays a key role in memory formation and learning (Spalding et al., 2013; Voss et al., 2017) and has been involved in spatial mapping and internalizing behaviors, such as anxiety and depression (Engin and Treit, 2007; Gatt et al., 2009; Gujral et al., 2017). In addition, the hippocampus is involved in control of hypothalamic-pituitary-adrenal (HPA) axis negative feedback (Snyder et al., 2011), and corticosteroid exposure seems to be linked to hippocampal volume and function (Sapolsky et al., 1990; Brown et al., 2004). Stressors occurring between the fetal period and childhood, as well as genetic factors [reviewed in Miguel et al. (2019)], can influence hippocampal development, thereby inducing long-term effects on brain structure and function.

Prenatal maternal stress (PNMS) is one such factor that has been thoroughly researched for its effects on hippocampal development in animals. These studies have provided substantial evidence that PNMS affects hippocampal development (Charil et al., 2010; Ortega-Martinez, 2015; Grigoryan and Segal, 2016). Magnetic resonance imaging (MRI) scans demonstrate that PNMS is associated with reduced hippocampal volume (Uno et al., 1994; Schmitz et al., 2002; Coe et al., 2003). Although these studies consistently demonstrate a reduction in hippocampal volume following PNMS, Schmitz et al. (2002) report a sex-specific effect, with reduced volume only observed in female rats, while other studies find effects in both sexes.

In humans, Qiu et al. (2013) have demonstrated that increased maternal anxiety during pregnancy was associated with reduced hippocampal growth in the offspring’s first 6 months of life, suggesting that maternal anxiety during pregnancy predicts differences in hippocampal development; however, it remains unclear whether it is the heritable trait of anxiety or exposure to maternal stress hormones in the intrauterine environment that precipitates this effect. Recent research yields inconsistent results, such that stressful life events during the prenatal or early postnatal period were not associated with hippocampal volume (Marečková et al., 2018). Many studies have also investigated the effect of postnatal stress on hippocampal structure in humans. It has been demonstrated that patients with posttraumatic stress disorder, as a result of adversity in early life or adulthood, exhibit reduced hippocampal volume (O’Doherty et al., 2015; Bromis et al., 2018). However, other research has failed to replicate previous findings (De Bellis et al., 1999; Bonne et al., 2001; Yamasue et al., 2003). For example, a study of Vietnam war veterans with PTSD and their combat-naïve identical twins suggests, in fact, that smaller hippocampal volumes represent a pre-existing risk factor for developing PTSD in the face of trauma (Shin et al., 2006). Taken together, the disparity in findings concerning the effect of stress on hippocampal structure in humans, and the different effects of PNMS in male and female animal models, suggests that individuals may be differentially susceptible to the effects of PNMS. Differential susceptibility can occur as a function of genetic differences in the population that alter an individual’s vulnerability to the effects of life events.

Indeed, single-nucleotide polymorphisms (SNPs) that affect brain development were observed to moderate the effect of maternal anxiety during pregnancy on the child’s brain structure. For example, a SNP rs6265 converting a valine (Val) to methionine (Met) on the brain-derived neurotrophic factor (BDNF) gene, which promotes the growth, maturation and survival of nerve cells, influences the degree to which maternal anxiety induces DNA methylation in the offspring, and influences the relationship between the offspring’s methylation and brain volume (Chen et al., 2015). Specifically, this paper reports that the Met/Met genotype in offspring was associated with a greater impact of maternal anxiety on DNA methylation and with a greater correlation between DNA methylation and right amygdala volume. Meanwhile, the Val/Val genotype was associated with a greater correlation between DNA methylation and left hippocampal volume. Moreover, another study reported that the interaction between the BDNF rs6265 Met allele and low family cohesion is associated with smaller left hippocampal volume in subjects with pediatric bipolar disorder (Zeni et al., 2016). Rabl et al. (2014) explored gene -by- environment effects between SNPs and adverse life events on hippocampal volume in healthy individuals. Among the SNPs studied, catechol-*O*-methyltransferase (COMT) Val158Met and BDNF Val66Met moderated the association between adverse life events and hippocampal volume in a large sample of healthy humans. The rs4680 COMT gene variant induces a Val to Met amino acid transition at codon 158 (Val158Met), resulting in a 4-fold decrease in enzyme activity in Met carriers. Qiu et al. (2015) investigated whether SNPs in the COMT gene of offspring could moderate the effects of maternal anxiety on brain structure, specifically prefrontal and parietal cortical thickness. The authors found that among rs737865-val158met-rs165599 haplotypes, the A-val-G haplotype exhibited a positive relationship between maternal anxiety and the offspring’s cortical thickness in the right ventrolateral prefrontal cortex and the right superior parietal cortex. Meanwhile, the G-met-A haplotype exhibited a negative relationship between maternal anxiety and the offspring’s cortical thickness in the bilateral precentral gyrus and dorsolateral prefrontal cortex. This demonstrates that particular COMT genotypes confer heightened vulnerability of frontal and parietal cortex regions to the effects of prenatal maternal anxiety. While these studies demonstrate that SNPs moderate the relationship between maternal anxiety and *in utero* neurodevelopment, further research is required to elucidate this relationship. The research to date has investigated only short-term effects of maternal anxiety on brain structure, demonstrating effects on children up to 6 months old.

Unlike animal studies, human research often lacks a randomly assigned stressor. The limitation of stressors from human studies is that they may be associated with an individual’s traits, such as impulsivity or neuroticism, which may be transmitted to the offspring genetically. If a pregnant woman experiences stress that she or the father may have induced, in part, by their own temperament (e.g., divorce or job loss), and their child grows up to develop a similarly difficult temperament, it becomes almost impossible to determine the extent to which the association between the stress in pregnancy and the child’s difficulties are due to genetic transmission, the intrauterine environment, and the postnatal rearing environment. Natural disasters randomly affect large populations and have a sudden onset that affects pregnant women in different stages of pregnancy. Project Ice Storm provides a unique opportunity to determine the effects of the mothers’ objective hardship, subjective distress and cognitive appraisal of a stressor through a quasi-randomly assigned event. Consequently, we use natural disasters to study the effects of prenatal maternal stress on the development of the offspring.

In the current project, studying PNMS derived a natural disaster, our goal was to (1) determine the effect of three measures of PNMS (objective hardship, subjective distress, and a negative cognitive appraisal) on left and right hippocampal volume in young adolescents; (2) determine the effect of genetic variants of COMT Val158Met and BDNF Val66Met on left and right hippocampal volume; and (3) determine the extent to which selected SNPs moderate the effect of PNMS on hippocampal volume. Since sex is an important determinant for the effects of stress on brain development, we expected that SNPs would differentially affect males and females.

## Materials and Methods

### Participants

#### Recruitment

Following the ice storm in January 1998, our research group contacted obstetricians in the Montérégie, a region southeast of Montreal, Canada that was highly affected by the crisis. Physicians from four hospitals in the region identified women who met the following criteria: (1) were pregnant during, or within 3 months of the ice storm; (2) French Canadian; and (3) 18 years old or older. The families that responded were significantly better educated and had higher incomes than the regional averages. Families who gave consent have been assessed periodically. The protocols were approved by the Research Ethics Board of the Douglas Hospital Research Centre.

#### Participants

In this study, 53 children who were *in utero* during the ice storm, or were conceived within 3 months of the ice storm, were assessed at the age of 11 1/2 years. Among the 53 children, 15 (28.3%) had been in their first, 14 (26.4%) in their second, and 10 (18.9%) in their third trimester on January 9, 1998 (the peak of the ice storm). The remaining 14 (26.4%) children were conceived within 3 months of the storm; they were also considered as “exposed” because maternal stress hormones could still be elevated within 3 months of a major stressor. The participants included 27 boys and 26 girls for whom both brain and genotype data were available. We included children who were conceived within 3 months following the ice storm (preconception) because of the potential for long-term effects of the ice storm which continued to affect the population after the reference date of January 9, 1998. All participants were right-handed.

### Measures

#### Prenatal Maternal Stress

Objective hardship, subjective distress, and cognitive appraisal measures were collected through maternal questionnaires mailed to the families on June 1, 1998, 5 months after the beginning of the ice storm.

##### Objective hardship

To estimate objective hardship of the mother, our group developed a questionnaire with items to evaluate four categories of exposure objectively (threat, loss, scope, and change). Questions in each category quantified experiences such as “number of days without electricity” and “number of displacements from home”. Each category has a maximum score of eight points and is summed to create the Storm32 score (Laplante et al., 2007). In our sample, the Storm32 scores ranged from 5 to 24 and averaged 11.55 (SD = 4.53).

##### Subjective distress

The subjective distress of mothers was evaluated using the 22-item Impact of Event Scale-Revised (IES-R) (Weiss and Marmar, 1997), which includes questions concerning the severity of posttraumatic stress-like symptoms in three categories (hyperarousal, intrusion, and avoidance). The IES-R has good internal consistency (α = 0.93) and satisfactory test-retest reliability (*r* = 0.76) (Brunet et al., 2003) and was adapted to relate specifically to the ice storm. A cutoff score of 33 is often used to screen for probable PTSD. In our sample, the IES-R scores ranged from 0 to 40 and averaged 9.43 (SD = 9.68).

##### Cognitive appraisal

The mother’s cognitive appraisal of the storm was assessed by asking “If you think about all of the consequences of the ice storm on your household members, would you say they were” and providing five response options on a Likert scale [“Very negative” (1), “Negative” (2), “None” (3), “Positive” (4), and “Very positive” (5)]. As our interest is the effect of negative cognitive appraisal about the ice storm on child outcomes, we compared the “Negative cognitive appraisal group” (recoded as 0), which included participants who had rated the consequences as very negative and negative, with a “Neutral/Positive cognitive appraisal group” (recoded as 1), which included participants who had rated the consequences as none, positive, or very positive.

#### Hippocampal Volume

##### MRI acquisition

Anatomical magnetic resonance imaging (MRI) was performed at the *Unité de Neuroimagerie Fonctionnelle (UNF) du Centre de Recherche de l’Institut Universitaire de Gériatrie de Montréal* (CRIUGM) on a 3.0T Siemens MAGNETOM Trio TIM Syngo (Siemens, Erlangen, Germany), with a 12-channel head coil. A total of 65 children underwent a three-dimensional, high-resolution, whole brain, structural T1-weighted magnetization-prepared gradient-echo image (MP-RAGE) sequence; TR = 2,300 ms, TE = 2.98 ms, TI = 900 ms; 256 mm field of view, 1 mm slice thickness, 176 slices, sagittal acquisition, time = 9 min. For this study, only 53 children’s data were analyzed, as rest of 12 children had no genetic data.

##### MRI preprocessing

All MR images were converted from their standard Digital Imaging and Communications in Medicine (DICOM) format to MINC2 (Medical Image NetCDF). Images were then corrected for intensity non-uniformity and underwent normalization for signal intensity (Talairach and Tournoux, 1988).

##### Total intracranial volume

For each subject, total intracranial volume (TIV) was automatically obtained using the Brain Extraction based on non-local Segmentation Techniques (BEaST) method (Eskildsen et al., 2012). The resulting skull masks were then manually corrected by an expert rater.

##### Automatic segmentation of the hippocampus

Bilateral hippocampal volumes, including subfields [(i) cornu ammonis (CA) 1, (ii) CA2/CA3, (iii) CA4/dentate gyrus, (iv) stratum radiatum/stratum lacunosum/stratum moleculare, and (v) subiculum] were automatically segmented using the Multiple Automatically Generated Templates brain segmentation (MAGeT-Brain) algorithm, which includes input from digital atlases by Winterburn et al. (2013), based on five high-resolution (0.3 mm isotropic) T1-weighted images (two males and three females, ages 29–57, avg. 37). The hippocampal atlases described here are available freely online^1^ and when used with the MAGeT-Brain^2^ segmentation technique produce reliable delineations of the hippocampus, including subfields.

##### Manual corrections and normalization

Whole left- and right-hippocampal volumes were delineated by merging the automated segmentation outputs of the hippocampal subfields to produce a single label for the whole hippocampus in each hemisphere. To increase the precision and validity of volumetric results, all hippocampal labels underwent manual corrections by an expert rater following the Pruessner segmentation protocol (Pruessner et al., 2000). Finally, to control for interindividual differences in total intracranial volume that may account for differences in hippocampus volume, hippocampal volumes were normalized by calculating a hippocampal volume-total intracranial volume ratio (HCV/TIV).

Table 1 presents descriptive statistics for all of the PNMS, demographic, and brain variables for boys and girls separately.

**TABLE 1:** Descriptive analysis of variables.

**Sex Child**	**Variables**	***N***	**Min**	**Max**	**Mean**	**SD**
Boys	Objective PNMS	27	5.00	24.00	11.2593	4.26608
	Subjective PNMS	27	0.00	40	10.2593	10.89316
	Maternal cognitive appraisal	27				
	Negative	7 (25.9%)				
	Positive	20 (74.1%)				
	Number of days of pregnancy when ice storm happened	27	−62	274	89.93	101.424
	Gestational age at birth (weeks)	27	32.29	41.29	39.4709	1.99009
	Edinburgh postnatal depression score	26	1.00	16.00	7.7308	4.01555
	Socioeconomic status (SES) Hollingshead scale	27	11.00	44.00	27.5926	10.39696
	Number of cigarettes/day	27	0.00	15.00	1.2593	3.85898
	Number of glasses of alcohol/week	27	0.00	2.00	0.1611	0.45454
	Child birth weight	27	1655.0	4185.0	3405.945	611.0644
	Left HCV	27	2302	3649	3002.48	339.067
	Right HCV	27	2310	3406	2953.37	296.586
	Left HCV_TIV RATIO	27	0.18	0.25	0.2125	0.01974
	Right HCV_TIV RATIO	27	0.18	0.23	0.2092	0.01825
	TIV	27	1243630	1603640	1412972.22	93490.764
Girls	Objective PNMS	26	5.00	24.00	11.8462	4.85545
	Subjective PNMS	26	0.00	24	8.5692	8.36712
	Maternal cognitive appraisal	26				
	Negative	11 (42.3%)				
	Positive	15 (57.7%)				
	Number of days of pregnancy when ice storm happened	26	−73	261	92.38	99.407
	Gestational age at birth (weeks)	26	32.86	41.43	39.4451	1.63997
	Edinburgh postnatal depression score	26	1.00	13.00	5.1538	3.42569
	Socioeconomic Status (SES) Hollingshead scale	26	11.00	65.00	26.9231	12.21449
	Number of cigarettes/day	26	0.00	25.00	2.1538	5.40698
	Number of glasses of alcohol/week	26	0.00	2.00	0.1182	0.43080
	Child birth weight	25	1855.0	4432.0	3426.486	535.0978
	Left HCV	26	2426	3322	2932.38	246.412
	Right HCV	26	2350	3201	2864.00	233.635
	Left HCV_TIV RATIO	26	0.18	0.27	0.2170	0.02025
	Right HCV_TIV RATIO	26	0.18	0.25	0.2120	0.02002
	TIV	26	1177870	1579900	1354365.38	83589.316

*HCV, hippocampal volume; TIV, total intracranial volume.*

#### Genotype Assessment

When Project Ice Storm children were 8 1/2 years old, saliva samples were collected during a laboratory assessment using Oragene DNA self-collection kit (OG-500) (DNA Genotek) and stored at room temperature until further analysis. DNA extraction was performed using PrepIT-L2P kit (DNA Genotek) according to the manufacturer’s instructions. DNA yield was measured using NanoDrop 8000 Spectrophotometer V2.1 (Thermo Fisher Scientific). DNA was stored at −80°C until analysis. rs6265 (BDNF) and rs4680 (COMT) were genotyped using Sequenom iPLEX Gold Technology (Ehrich et al., 2005) at McGill University and the Génome Québec Innovation Centre. All participants had call rates >98%, indicating generally good quality DNA and results.

#### Genotype Frequencies

The major/major homozygote, heterozygote, and minor/minor homozygote genotype frequencies of each SNP are presented in Table 2. In addition, we tested each SNP for accordance with the Hardy-Weinberg equilibrium, which indicates whether the genotype frequencies in our sample were representative of the general population. Indeed, the SNP located on COMT met the Hardy-Weinberg equilibrium; however, the SNP located on BDNF could not be tested for the Hardy-Weinberg equilibrium because the minor genotype was not represented. Although studying BDNF using our sample was, therefore, limited, we still assessed the effect of having 1 or 2 major BDNF alleles on hippocampal volume because of the importance of this gene in hippocampal development. SNP genotype coding for COMT for the three different comparisons was as follows: AA (1) vs. AG (2) vs. GG (3); AA (1) vs. AG + GG (2); GG (1) vs. AA + AG (2). Genotype coding for the single BDNF comparison: CC (1) vs. TC (2).

**TABLE 2:** Genotype frequencies.

	**Boys**			**Girls**		
	**Major/Major homozygote**	**Heterozygote**	**Minor/Minor homozygote**	**Major/Major homozygote**	**Heterozygote**	**Minor/Minor homozygote**
COMT rs4680	GG; Val/Val 3 (11.1%)	GA; Val/Met 20 (74.1%)	AA; Met/Met 4 (14.8%)	GG; Val/Val 5 (19.2%)	GA; Val/Met 8 (30.8%)	AA; Met/Met 13 (50.0%)
BDNF rs6265	CC; Val/Val 9 (33.3%)	CT; Val/Met 18 (66.7%)	TT not represented	CC; Val/Val 4 (15.4%)	CT; Val/Met 22 (84.6%)	TT not represented

*Frequency is represented in number of participants and percent of participants. For BDNF, the minor homozygous Met/Met genotype was not represented in our sample.*

#### Control Variables

Many factors are known to alter fetal development, and thus, induce long-term effects on hippocampal volume. To control for this, we tested the effect of some of these factors (maternal cigarette smoking during pregnancy, maternal alcohol use during pregnancy, socioeconomic status, birth weight, and timing of exposure during pregnancy) on HCV/TIV ratio in boys and girls. We controlled for factors that were correlated with HCV/TIV. The correlations between these potential control variables and HCV/TIV are presented in Table 3.

**TABLE 3:** Associations between risk factors and HCV/TIV ratios in boys and girls.

	**Boys**		**Girls**	
	**Left HCV/TIV ratio**	**Right HCV/TIV ratio**	**Left HCV/TIC ratio**	**Right HCV/TIV ratio**
Mother’s cigarettes during pregnancy	*r* = −0.179	*r* = −0.202	*r* = −0.151	*r* = −0.314
Maternal alcohol during pregnancy	*r* = −0.389*	*r* = −0.418*	*r* = 0.155	*r* = 0.313
Socioeconomic status	*r* = −0.333^#^	*r* = −0.281	*r* = 0.449*	*r* = 0.247
Birth weight	*r* = 0.309	*r* = 0.278	*r* = −0.040	*r* = −0.014
Timing of exposure during pregnancy	*r* = 0.062	*r* = 0.047	*r* = 0.121	*r* = 0.106

r, Pearson’s correlation coefficient; ^#^p < 0.10, *p < 0.05.

### Statistical Analysis

Correlations and ANOVAs were conducted to determine the associations between PNMS and SNPs on HCV/TIV ratio. Moderation hierarchical regression analyses were conducted to determine whether either of the two SNPs moderate the effect of PNMS on HCV/TIV ratio. More specifically, analyses tested whether the two SNPs of interest moderate the effect of objective hardship, subjective distress, and/or cognitive appraisal on left and right HCV/TIV ratios. First, as mentioned above (Table 3), potential covariates that correlated significantly with the outcome were entered in the model. Accordingly, when testing for effects in both left and right HCV/TIV ratio in boys, we controlled for the number of glasses of alcohol the mother drank per week during her pregnancy. In addition, for left HCV/TIV ratio in girls we controlled for socioeconomic status (SES). No potential covariates were added to the right HCV/TIV model in girls. Additionally, for analyses focusing on subjective distress or cognitive appraisal, objective hardship was included as a covariate. Second, one of the components of prenatal stress (objective hardship or subjective distress or cognitive appraisal) was entered. Third, genotypes (COMT or BDNF) were entered: analyses included the number of major or minor alleles, but also dichotomized variables such that genotype groups included either minor allele carriers (minor merged with heterozygote) vs. major/major genotype carriers, or major allele carriers (major merged with heterozygote) vs. minor/minor genotype carriers. Finally, the PNMS-by-SNP interaction term was added to the model. All analyses were conducted for boys and girls separately.

## Results

### Bivariate Correlations Between PNMS or Genotype and HCV/TIV Ratio

As shown in Table 4, in boys, there was a marginally significant correlation between greater objective hardship and larger left HCV/TIV ratio (*r* = 0.324, *p* = 0.099). In girls, objective hardship was positively correlated with right HCV/TIV ratio (*r* = 0.392, *p* = 0.048). Neither subjective distress nor cognitive appraisal was significantly associated with HCV/TIV ratio in girls or boys.

**TABLE 4:** Associations between PNMS and genotypes with HCV/TIV ratios in boys and girls.

	**Boys**		**Girls**	
	**Left HCV/TIV ratio**	**Right HCV/TIV ratio**	**Left HCV/TIV ratio**	**Right HCV/TIV ratio**
Objective PNMS	*r* = 0.324^#^	*r* = 0.280	*r* = 0.305	*r* = 0.392*
Subjective PNMS	*r* = −0.014	*r* = 0.317	*r* = 0.142	*r* = 0.023
Cognitive appraisal	*r* = −0.093	*r* = 0.032	*r* = 0.103	*r* = −0.019
COMT (rs4680)	*r* = 0.561**	*r* = 0.334^#^	*r* = −0.099	*r* = −0.259
BDNF (rs6265)	*r* = 0.369^#^	*r* = 0.160	*r* = −0.457*	*r* = −0.348^#^

*HCV, hippocampal volume; TIV, total intracranial volume.*

r, Pearson’s correlation coefficient; ^#^p < 0.10, *p < 0.05, **p < 0.01.

*Genotype coding:*

*COMT: AA = 1, AG = 2, GG = 3.*

*BDNF: CC = 1, TC = 2.*

**FIGURE 1:**
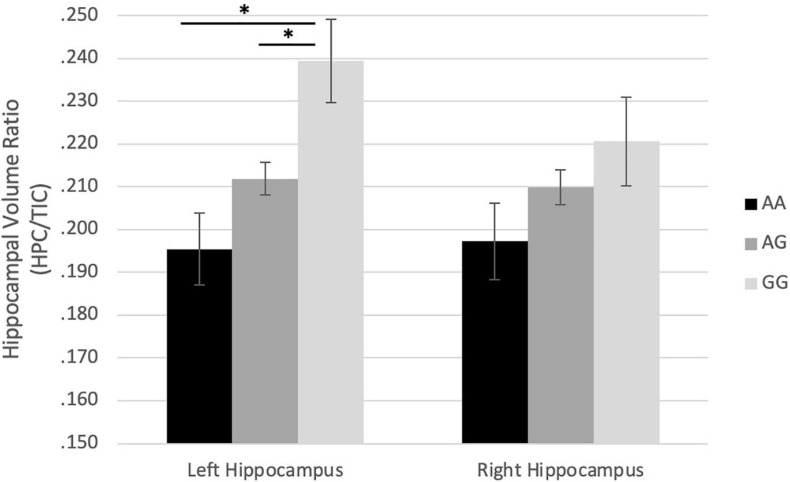
Effect of rs4680 (COMT) genotype on hippocampus/TIC ratio volume in boys. ^∗^*p* < 0.05.

In boys, the COMT genotypes were correlated with left HCV/TIV ratio (*r* = 0.561, *p* = 0.002) with a similar trend on the right HCV/TIV ratio (*r* = 0.334, *p* = 0.089) (Table 4). An ANOVA (*p* = 0.008, *F* = 5.917, df = 2) and Tukey *post hoc* test demonstrated that in boys the major homozygote genotype (GG) was significantly associated with larger left HCV/TIV ratio compared to the heterozygote (GA) (*p* = 0.037) and that of the minor homozygote (AA) (Figure 1). For BDNF genes, there was a trend for the major homozygote (CC) of the BDNF gene to be associated with smaller Left HCV/TIV ratio (*r* = 0.369, *p* = 0.058) in boys (Table 4), however, no significant finding for right HCV/TIV ratio was observed.

**FIGURE 2:**
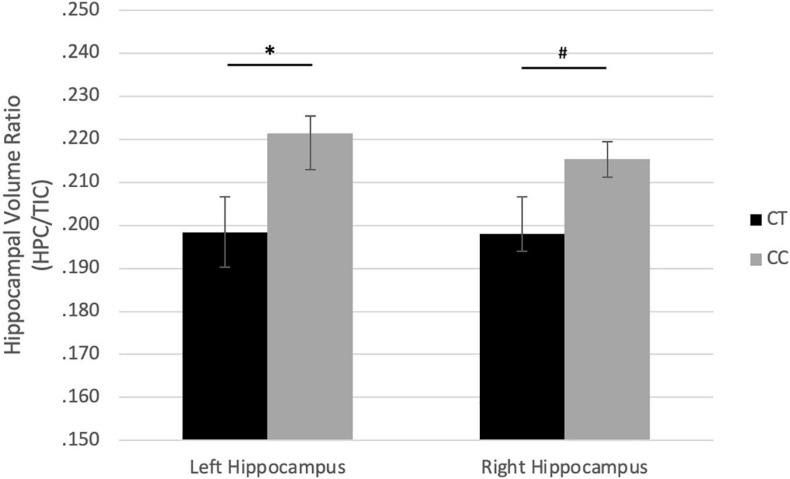
Effect of rs6265 (BDNF) genotype on hippocampus/TIC ratio volume in girls. ^#^*p* < 0.10, ^∗^*p* < 0.05.

In girls, there were no associations between COMT genotypes and HCV/TIV. For BDNF genotypes, the major homozygote genotype (CC) associated with larger left HCV/TIV ratio (*r* = −0.457, *p* = 0.019), with a similar trend with right HCV/TIV (*r* = −0.348, *p* = 0.082) (Figure 2).

### Gene-by-Environment Interaction

Moderation hierarchical regression analyses were conducted to test whether the two SNPs of interest moderate the effect of objective hardship, subjective distress and cognitive appraisal on the ratio of left and right HCV/TIV ratio.

#### Effect of PNMS, Genotype and Gene-by-Environment Interaction on HCV/TIV Volume in Boys

##### For the left HCV/TIV ratio in boys

In the objective hardship model, we found that the covariate, maternal alcohol consumption during pregnancy, explained 15.1% (*p* = 0.045) of variance in the left HCV/TIV. Next, a marginally significant 9.3% of additional variance was explained by objective hardship levels (*p* = 0.099): greater hardship was associated with larger left HCV/TIV (Table 5). The addition of the main effects of the genotypes increased variance explained by 16.3% (*p* = 0.020) with significant effects from COMT genotypes [major homozygotes (GG) vs. heterozygotes (GA) vs. minor homozygotes (AA)], or 14.9% (*p* = 0.026) additional variance explained when comparing effects from major homozygotes (GG) vs. minor allele carriers (AA + AG). The full models explained 40.6 and 39.2% of variance, respectively. The main effect of minor homozygotes (AA) vs. major allele carriers (AG + GG) and the interactions were not significant.

**TABLE 5:** Effect of PNMS, genotype and gene-by-environment interaction on HCV/TIV volume in boys, controlling for maternal alcohol consumption during pregnancy with left and right HCV/TIV volume.

	**Left HCV/TIV ratio (Boys)**										**Right HCV/TIV ratio (Boys)**									
	**Objective PNMS**			**Genotype**			**Interaction**			**Total**	**Objective PNMS**			**Genotype**			**Interaction**			**Total**
	**BETA^a^**	**CH *R*^2^**	***p***	**BETA**	**CH *R*^2^**	***p***	**BETA**	**CH *R*^2^**	***p***	***R* ^2^**	**BETA^a^**	**CH *R*^2^**	***p***	**BETA**	**CH *R*^2^**	***p***	**BETA**	**CH *R*^2^**	***p***	***R* ^2^**
**COMT**																				
*AA vs. AG vs. GG*				0.437	0.163	**0.020**	1.586	0.032	0.272	0.439				0.170	0.025	0.387	−2.101	0.057	0.188	0.324
*AA* + *AG vs. GG*	0.305	0.093	0.099	−0.399	0.149	**0.026**	−1.422	0.014	0.473	0.407	0.259	0.067	0.158	−0.121	0.014	0.521	4.154	0.122	**0.049**	0.378
*AA vs. AG* + *GG*				0.233	0.047	0.228	0.626	0.002	0.817	0.293				0.124	0.013	0.527	−1.676	0.013	0.545	0.268

	**Subjective PNMS**			**Genotype**			**Interaction**			**Total**	**Subjective PNMS**			**Genotype**			**Interaction**			**Total**
	**BETA^b^**	**CH *R*^2^**	***p***	**BETA**	**CH *R*^2^**	***p***	**BETA**	**CH *R*^2^**	***p***	***R* ^2^**	**BETA^b^**	**CH *R*^2^**	***p***	**BETA**	**CH *R*^2^**	***p***	**BETA**	**CH *R*^2^**	***p***	***R* ^2^**

**COMT**																				
*AA vs. AG vs. GG*				0.426	0.153	**0.025**	−0.026	0.000	0.970	0.414				0.201	0.034	0.301	0.716	0.032	0.318	0.363
*AA* + *AG vs. GG*	−0.142	0.018	0.465	−0.388	0.138	**0.035**	0.293	0.003	0.731	0.403	0.250	0.055	0.192	−0.154	0.022	0.411	−0.958	0.037	0.285	0.356
*AA vs. AG* + *GG*				0.227	0.045	0.246	0.428	0.005	0.691	0.312				0.135	0.016	0.484	0.559	0.009	0.602	0.322

	**Cognitive appraisal**			**Genotype**			**Interaction**			**Total**	**Cognitive appraisal**			**Genotype**			**Interaction**			**Total**
	**BETA^b^**	**CH *R*^2^**	***p***	**BETA**	**CH *R*^2^**	***p***	**BETA**	**CH *R*^2^**	***p***	***R* ^2^**	**BETA^b^**	**CH *R*^2^**	***p***	**BETA**	**CH *R*^2^**	***p***	**BETA**	**CH *R*^2^**	***p***	***R* ^2^**

**COMT**																				
*AA vs. AG vs. GG*	0.095	0.007	0.654	0.453	0.173	**0.018**	−1.187	0.043	0.210	0.466	0.235	0.041	0.265	0.199	0.033	0.312	1.617	0.079	0.113	0.395
*AA* + *AG vs. GG*				−0.395	0.145	**0.031**	0.897	0.023	0.375	0.418				−0.107	0.011	0.570	−1.847	0.096	0.083	0.390

*CH R^2^, Change in R-squared (R^2^).*

*a: control for the number of glasses of alcohol the mother drank per week during her pregnancy.*

*b: control for the number of glasses of alcohol the mother drank per week during her pregnancy, objective PNMS.*

*COMT genotype coding:*

*AA (1) vs. AG (2) vs. GG (3);*

*AA (1) vs. AG + GG (2);*

*GG (1) vs. AA + AG (2);*

*Bold means significant.*

In the subjective distress model, maternal alcohol consumption and objective hardship explained a significant 24.4% of variance in the outcome (*p* = 0.035). Subjective distress explained a non-significant additional 1.8% of variance. The main effects of the COMT genotypes [major homozygotes (GG) vs. heterozygotes (GA) vs. minor homozygotes (AA)] significantly increased variance explained by 15.3% (*p* = 0.025) while a significant additional 13.8% of the variance (*p* = 0.035) was explained by the major homozygotes (GG) vs. minor-allele carriers (AA + AG) genotypes. The full models explained 41.4 and 40.0% of variance, respectively. The main effect of minor homozygotes (AA) vs. major allele carriers (AG + GG) as well as the interactions were not significant.

In the cognitive appraisal model, maternal alcohol consumption during pregnancy and objective hardship explained a significant 24.4% of variance in the outcome (*p* = 0.035). Cognitive appraisal explained a non-significant additional 0.7% of variance. The COMT genotype main effect (major homozygote vs. heterozygote vs. minor homozygote) significantly increased variance explained by 17.3% (*p* = 0.018), while a significant additional 14.5% of the variance was explained by the comparison of the minor homozygotes vs. major-allele carriers (*p* = 0.031). The full models explained 42.3 and 39.6% of variance, respectively. The main effect of minor homozygotes (AA) vs. major allele carriers (AG + GG) as well as the interactions were not significant.

##### For the right HCV/TIV ratio in boys

**FIGURE 3:**
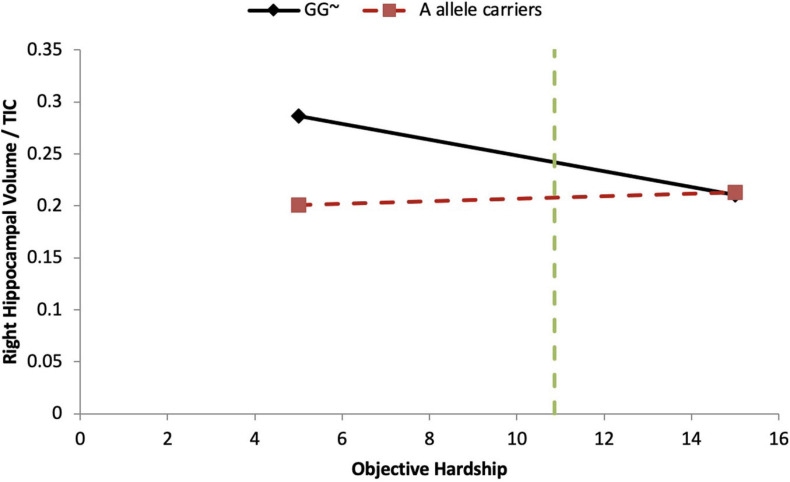
COMT moderates objective hardship on right HCV/TIV ratio in boys. Moderation analyses demonstrate that there is a significant COMT-by-Objective PNMS interaction effect in boys (*p* = 0.049). For the major homozygote (COMT genotype GG, solid line) there is a marginally significant negative association between objective hardship and right HCV/TIV ratio volume (*p* = 0.084); however, for the heterozygote and minor homozygote COMT genotypes (A allele carriers, red dashed line) there is no association between objective hardship and right HCV/TIV ratio. There is a region of significance (*p* < 0.05) when objective hardship levels are below 10.86, such that participants with major genotypes have a larger right HCV/TIV ratio compared to minor allele carriers. The green dashed line indicates the value of 10.83. ∼*p* < 0.10.

In the objective hardship model, we found that the covariate, maternal alcohol consumption during pregnancy, explained 17.5% (*p* = 0.03) of the variance. Neither the main effect of objective hardship (6.7%) nor of COMT genotypes (1.3–2.5%) were significantly associated with right HCV/TIV. However, COMT genotypes [major homozygotes (GG) vs. minor-allele carriers (AA + AG)] were found to significantly moderate the effect of objective hardship on right HCV/TIV ratio (*R*^2^-Change = 0.122, *p* = 0.049), with the full model explaining 37.8% of the variance in right HCV/TIV volume (Table 5). As shown in Figure 3, for major homozygotes (GG), there was a negative association between objective hardship and right HCV/TIV volume (*p* = 0.084) with greater objective hardship associated with smaller volumes; however, for minor-allele carriers (GA and AA), there was no association between objective hardship and right HCV/TIV volume (*p* = 0.106). Below objective hardship levels of 10.86 (slightly below the group average) there was a significant difference in right HCV/TIV volume between genotypes, with major homozygotes having larger volumes than the minor-allele carriers. At low levels of maternal objective hardship, the mean right HCV of GG boys is approximately 4 standard deviations (SD) above the group mean while at moderate levels of hardship the GG and A-allele carriers all averaged right HCV at the group mean. The other interactions with objective hardship were not significant.

The main effects of subjective distress or cognitive appraisal on right HCV/TIV volume, or the interactions including them, were not significant.

Thus, in boys, the only significant interaction effect between any maternal stress components and genotypes was between objective hardship and COMT on right HCV/TIV ratio. The full models, including maternal stress, COMT genotype, G × E interactions (if significant), and the covariate maternal alcohol consumption during pregnancy, explained 25.5–42.3% of the variance.

There were no significant interaction effects between PNMS and BDNF genotypes on hippocampus volumes in boys.

#### Effect of PNMS, Genotype and Gene-by-Environment Interaction on HCV/TIC Volume in Girls

##### For the left HCV/TIV ratio in girls

In the objective hardship model the covariate, SES, explained 20.1% (*p* = 0.021) of variance. There were no significant main effects of objective maternal stress (0.8%) nor of COMT genotypes (<0.1–0.7%), and no significant interaction effects.

**FIGURE 4:**
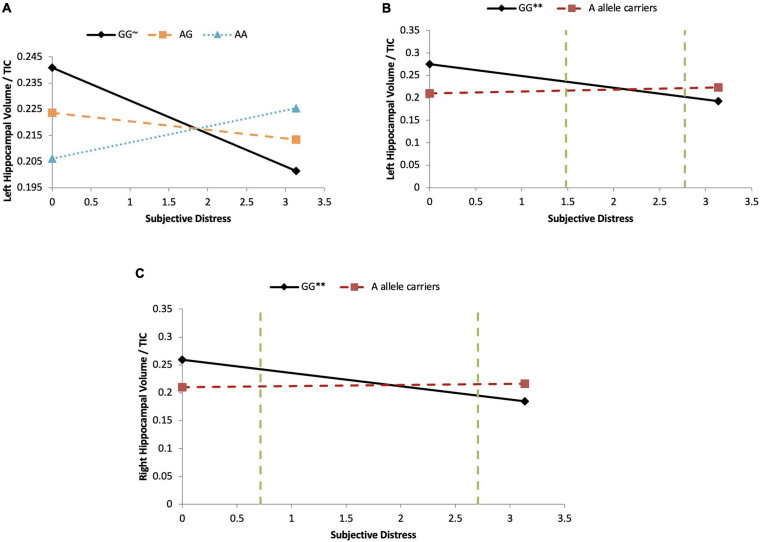
COMT moderates subjective distress on left HCV/TTV ratio in girls. **(A)** *Major homozygotes vs. heterozygotes vs. minor homozygotes:* Moderation analyses demonstrate that there is a significant COMT-by-subjective distress interaction effect on left HCV/TIV ratio in girls (*p* = 0.047). For the minor homozygote (AA genotype, dotted line) and heterozygote (AG genotype, dashed line) there is no association between subjective distress and left HCV/TIV ratio (*p* = 0.165 and *p* = 0.409, respectively). For major homozygote (GG genotype, solid line) there is a marginally significant negative association between subjective distress and left HCV/TIV ratios (*p* = 0.092). There are no statistical significance transition points within the observed range of the predictor. ∼*p* < 0.10. **(B)** *Major homozygotes vs. minor allele carriers on left HCV/TIV ratio:* Moderation analyses demonstrate that there is a significant COMT-by- subjective distress interaction effect on left HCV/TIC ratio in girls (*p* = 0.001). For major homozygotes (GG genotype, solid line), there is a significant negative association between subjective distress and left HCV/TIV ratio (*p* = 0.0026) but for minor homozygotes and heterozygotes (AA and AG genotypes, red dashed line) there is no association between subjective distress and left HPV/TIV ratio (*p* = 0.129). There are regions of significance (*p* < 0.05) such that below log-transformed subjective distress levels of 1.48 and above 2.77 there is a significant difference between left HCV/TIV ratio of major homozygotes and minor-allele carriers. ^∗∗^*p* < 0.01. **(C)** *Major homozygotes vs. minor allele carriers on right HCV/TIV ratio:* Moderation analyses demonstrate that there is a significant COMT-by-subjective distress interaction effect on right HCV/TIC ratio in girls (*p* = 0.007). For major homozygotes (GG genotype, solid line) there is a significant association between subjective distress and right HPV/TIV ratio volume (*p* = 0.008); however, for minor homozygotes and heterozygotes (AA and AG genotypes, red dashed line) there is no significant association between subjective distress and HCV/TIV ratio (*p* = 0.272). There is a region of significance (*p* < 0.05) between genotypes below log-transformed subjective distress levels of 0.717 and above 2.705, such that right HCV/TIC ratio were significantly different between the major homozygotes and the minor-allele carriers. The two green dashed lines indicates the regions of significance. ^∗∗^*p* < 0.01.

In the subjective hardship model, SES and objective hardship explained together a marginally significant 21.0% of variance in the outcome (*p* = 0.067). Controlling for these covariates, subjective distress explained a non-significant additional 0.1% of variance, and COMT genotypes main effect were also non-significant (<0.1–0.7%). However, the COMT genotypes [major homozygotes (GG) vs. heterozygotes (GA) vs. minor homozygotes (AA)] were found to significantly moderate the effect of subjective distress on left HCV/TIV ratio (*R*^2^-Change = 0.144, *p* = 0.047), with the full model explaining 35.8% of the variance in the outcome (Table 6). As shown in Figure 4A, for major homozygotes (GG), there was a marginally significant negative association between subjective distress and left HCV/TIV ratios (*p* = 0.092) with greater subjective distress related to smaller volumes. Left HCV/TIV ratio is approximately 1 SD above the mean at low levels of maternal subjective distress but at high levels of distress (a log-IESR of 3.2 which is equivalent to an IES-R score of 23.5 indicative of possible PTSD) they are about 1 SD below the mean. For minor homozygotes or heterozygotes, however, there was no association between subjective distress and left HCV/TIV ratios. There is no statistical region of significance within the observed range. The COMT comparison of major homozygotes (GG) vs. minor-allele carriers (AA + AG) (Figure 4B) was also found to significantly moderate the effect of subjective distress on left HCV/TIV ratio in girls (*R*^2^-Change = 0.330, *p* = 0.001), with the full model explaining 54.1% of the variance in left HCV/TIV volume (Table 6). As shown in Figure 4B, for major homozygotes (GG), there was a significant negative association between subjective distress and left HCV/TIV ratio (*p* = 0.0026) with greater subjective distress related to smaller volumes; at low maternal subjective distress, the left HCV/TIV ratio for GG homozygotes was about 3 SD above the mean, while at high levels it was about 1.3 SD below the group mean. However, for minor-allele carriers, there was no association between subjective distress and left HCV/TIV ratios which remained at about the group mean level. Below log-transformed subjective distress level of 1.480 (equivalent to a low IES-R score of 3.39) there was a significant difference in left HCV/TIV volume between genotypes, with major homozygotes having larger volumes than the minor-allele carriers. However, above log-transformed subjective distress level of 2.771 (equivalent of a low-moderate IES-R score of 14.97) major homozygotes had significantly smaller volumes than the minor-allele carriers. The interaction with minor homozygotes vs. major allele carrier was not significant.

**TABLE 6:** Effect of PNMS, genotype and gene-by-environment interaction on HCV/TIC volume in girls, controlling for socioeconomic status With left HCV/TIV.

	**Left HCV/TIV ratio (Girls)**										**Right HCV/TIV ratio (Girls)**									
	**Objective PNMS**			**Genotype**			**Interaction**			**Total**	**Objective PNMS**			**Genotype**			**Interaction**			**Total**
	**BETA**	**CH *R*^2^**	***p***	**BETA**	**CH *R*^2^**	***p***	**BETA**	**CH *R*^2^**	***p***	***R* ^2^**	**BETA**	**CH *R*^2^**	***p***	**BETA**	**CH *R*^2^**	***p***	**BETA**	**CH *R*^2^**	***p***	***R* ^2^**
**COMT**																				
*AA vs. AG vs. GG*				−0.055	0.003	0.777	−0.467	0.020	0.468	0.233				−0.227	0.051	0.235	−0.623	0.036	0.320	0.241
*AA* + *AG vs. GG*	0.106	0.008	0.628	0.002	0.000	0.991	0.653	0.014	0.544	0.224	0.392	0.154	**0.048**	0.122	0.015	0.529	0.791	0.021	0.462	0.189
*AA vs. AG* + *GG*				−0.084	0.007	0.664	−0.527	0.020	0.461	0.237				−0.260	0.066	0.177	−0.816	0.049	0.235	0.269

	**Subjective PNMS**			**Genotype**			**Interaction**			**Total**	**Subjective PNMS**			**Genotype**			**Interaction**			**Total**
	**BETA**	**CH *R*^2^**	***p***	**BETA**	**CH *R*^2^**	***p***	**BETA**	**CH *R*^2^**	***p***	***R* ^2^**	**BETA**	**CH *R*^2^**	***p***	**BETA**	**CH *R*^2^**	***p***	**BETA**	**CH *R*^2^**	***p***	***R* ^2^**

**COMT**																				
*AA vs. AG vs. GG*				−0.058	0.003	0.769	−1.211	0.144	**0.047**	0.358				−0.220	0.048	0.261	−1.091	0.117	0.070	0.327
*AA* + *AG vs. GG*	0.031	0.001	0.879	0.009	0.000	0.966	3.271	0.330	**0.001**	0.541	−0.098	0.009	0.628	0.108	0.011	0.591	2.785	0.244	**0.007**	0.418
*AA vs. AG* + *GG*				−0.084	0.007	0.671	−0.640	0.035	0.347	0.252				−0.260	0.066	0.185	−0.738	0.047	0.257	0.275

	**Cognitive appraisal**			**Genotype**			**Interaction**			**Total**	**Cognitive appraisal**			**Genotype**			**Interaction**			**Total**
	**BETA**	**CH *R*^2^**	***p***	**BETA**	**CH *R*^2^**	***p***	**BETA**	**CH *R*^2^**	***p***	***R* ^2^**	**BETA**	**CH *R*^2^**	***p***	**BETA**	**CH *R*^2^**	***p***	**BETA**	**CH *R*^2^**	***p***	***R* ^2^**

**COMT**																				
*AA vs. AG vs. GG*				−0.090	0.008	0.637	0.335	0.009	0.618	0.286				−0.247	0.059	0.209	0.739	0.045	0.270	0.268
*AA* + *AG vs. GG*	0.255	0.059	0.197	0.061	0.003	0.759	−0.120	0.000	0.916	0.272	0.104	0.010	0.608	0.154	0.022	0.444	0.124	0.000	0.915	0.186
*AA vs. AG* + *GG*				−0.095	0.009	0.621	0.414	0.012	0.573	0.289				−0.264	0.068	0.178	1.062	0.077	0.141	0.308

*CH R^2^, Change in R-squared (R^2^).*

*COMT genotype coding:*

*AA (1) vs. AG (2) vs. GG (3);*

*AA (1) vs. AG + GG (2);*

*GG (1) vs. AA + AG (2);*

*Bold means significant.*

Controlling for SES and objective hardship, the main effect of cognitive appraisal and its interactions with COMT genotypes were not significant.

##### For the right HCV/TIV ratio in girls

In the objective hardship model, objective hardship had a significant main effect on right HCV/TIV ratio, such that higher PNMS was associated with a larger ratio, explaining an additional 15.4% of the variance (*p* = 0.048). However, there was no significant main effect of any genotypes on right HCV/TIV volumes (*R*^2^-change = 1.5–6.6%), or of any interactions involving objective hardship.

Controlling for objective hardship, the effect of subjective distress (*R*^2^-change = 0.9%) and COMT genotypes (*R*^2^-change = 1.1–6.6%) on right HCV/TIV volumes were not significant. However, COMT genotypes [major homozygotes (GG) vs. minor-allele carriers (AA + AG)] moderated the effect of subjective distress on right HCV/TIV ratios (*R*^2^-change = 0.244, *p* = 0.007) with the full model explaining 41.8% of the variance in the outcome (Table 6). As shown in Figure 4C, for major homozygotes (GG), there was a significant negative association between subjective distress and right HCV/TIV ratios (*p* = 0.008) with greater subjective distress related to smaller volumes; at low subjective distress GG homozygotes had right HCV/TIV ratios about 1.25 SD above the group mean while at high levels their HCV/TIV ratio was about 1.6 SD below the mean. For minor-allele carriers (AA + AG), however, there was no association between subjective distress and right HCV/TIV ratios. Below subjective distress level of 0.717 (equivalent to a very low IES-R score of 1.05) major homozygotes (GG) had significantly larger volumes than the minor-allele carriers (AA + AG), and above subjective distress level of 2.705 (equivalent to a low-moderate IES-R score of 13.95) major homozygotes (GG) had significantly smaller volumes than the minor-allele carriers (AA + AG). The other moderations involving subjective distress were not significant.

There were no significant main effect of cognitive appraisal or interactions involving this variable.

Thus, in girls, the significant interaction effects between maternal stress components and genotypes were between subjective distress and COMT on left and right HCV/TIV ratio. The full models, including maternal stress, COMT genotype, G × E interactions (if significant), and SES for the HCV/TIC ratio, explained 16.8–54.1% of the variance.

Additionally, there were no significant interaction effects between PNMS and BDNF genotypes on hippocampus volume in girls.

## Discussion

The main objective of this study was to test whether children’s genotype would moderate the effects of maternal stress derived from 1998 Quebec ice storm during pregnancy on their hippocampal volume, thereby resulting in differential effects of PNMS on brain structure.

First, we examined the main effect of each component of PNMS on hippocampal volume. The previous literature reported associations between increased prenatal maternal anxiety and reduced hippocampal growth (Qiu et al., 2013), and the non-association between perinatal stressful life events and hippocampal volume (Marečková et al., 2018). Surprisingly, then, we found that higher maternal objective hardship was associated with larger, not smaller, right hippocampal volume in girls. Therefore, it seems that different types of stressors during pregnancy could result in mixed findings, at least when not taking genotype into account.

Second, zero-order correlations revealed associations between genotypes and hippocampal volume with COMT (rs4680) and BDNF (rs6265). Major homozygotes of COMT (Val/Val) are associated with larger hippocampal volume in boys, while major homozygotes of BDNF (Val/Val) are associated with larger hippocampal volume in girls. Although COMT Val carriers were reported in previous studies to have smaller hippocampal volumes than Met allele carriers in healthy individuals (Giordano et al., 1993; Taylor et al., 2007), our observation is consistent with the recent finding that COMT Val allele was significantly associated with larger hippocampal volume in healthy Chinese college students (ages from 19 to 21 years) (Wang et al., 2013). These correlations do not, of course, take prenatal stress into account.

The only significant gene-by-environment interactions involved the COMT genotype. Under maternal objective hardship conditions that are below the group mean (Figure 3), boys with the COMT major homozygotes have more than one standard deviation greater right hippocampal volume than that of minor-allele carriers who have approximately average ratios; however, because the right HCV/TIV ratios of boys with major homozygotes decrease as objective hardship increases (while those of the minor allele carriers are unaffected by PNMS), under high objective hardship there is little difference in HCV/TIV ratios between COMT genotypes. COMT is highly expressed in the hippocampus and the COMT minor allele results in reduced COMT enzymatic activity 3–4 fold which leads to higher dopamine levels. The HPA axis is activated in response to stress, which results in the release of the cortisol. Although Project Ice Storm tends to have very low correlations between objective hardship and maternal cortisol levels at recruitment (5 months after the start of the storm), our finding might suggest that high acute cortisol levels at the time of the storm (that would have been triggered by stress) and lower dopamine availability in Val carriers due to the higher enzymatic activity may collaborate to negatively affect hippocampal volume in boys. Similarly, in girls at low levels of maternal subjective distress, girls with major homozygotes again exhibit about one standard deviation greater left and right hippocampal volumes than those associated with minor allele carriers. However, this group difference is not seen under conditions of low-medium subjective distress, and is reversed in both the left and right hippocampi under conditions of higher subjective distress (Figures 4B,C). Our finding is supported by a recent PTSD study in which COMT Val158Met polymorphism moderated the relationship between PTSD symptom severity and hippocampal volume (Hayes et al., 2017): reduced left hippocampal volume was observed with increasing PTSD symptom severity in Val/Val carriers, but there was no association between PTSD symptom severity and left hippocampal volume for either of the Met allele carriers, and there were no significant main effects, nor an interaction effect, for the right hippocampus. Further, the authors suggested that putatively lower dopamine availability in Val carriers may interact with traumatic stress to negatively affect hippocampal structure.

Remarkably, in our study adolescents in the major homozygote group have a significantly larger hippocampal volume in low objective and low subjective PNMS conditions compared to high PNMS conditions, while heterozygotes and minor homozygotes do not seem to be affected by the levels of PNMS. Therefore, our results suggest that as maternal stress increases in severity children who are carriers of the Val/Val genotype are more likely to show reductions in hippocampal volume than Met carriers, indicating that the major homozygotes may be more susceptible to the effects of PNMS.

In contrast to the sex differences in the effects of the COMT and BDNF genotypes on hippocampal volume at early adolescence, it has previously been reported that the minor homozygote BDNF genotype is associated with reduced hippocampal volume in *both* male and female adults (Bueller et al., 2006). Given that we measured hippocampal volume at 11 1/2 years old, rather than in adulthood, it is possible that the genotype-dependent sex differences in male and female hippocampal volume are specific to this age group, which represents the onset of adolescence. The mechanism underlying the sex-specific effects of COMT and BDNF SNPs on hippocampal volume in early adolescence remains unclear but is likely influenced by sex hormones that are highly expressed in the hippocampus and interact with hippocampal function (Goldstein et al., 2001). Sex hormones may influence the role of BDNF and COMT through various mechanisms. For example, estrogen is known to alter levels of BDNF expression, thereby leading to disproportionate regulation of BDNF protein levels in females compared to males (Sohrabji and Lewis, 2006). These same sex hormone differences found between adolescent males and females may also explain observed sex differences in the moderation effects. While the COMT genotype moderated the effect of prenatal maternal objective hardship in boys, this SNP moderated the effect of maternal subjective distress in girls. These sex-specific results are in line with our original hypothesis, which postulated that results would differ between males and females and indicate sex differences in brain development that are particularly prevalent at the onset of puberty. Our finding was supported by Jahnke et al. (2021) in which maternal stress during pregnancy was reported to influence the functioning of HSD11B2 in placenta in a sex-specific manner, suggesting that maternal chronic stress may exhaust HSD11B2’s protective mechanism, exposing the newborn to high amounts of maternal cortisol, which could alter the fetal HPA axis and influence long-term neurobehavioral development.

### Limitations and Advantages

Although the results described here further our understanding of the interaction between genetic variants and PNMS on hippocampal structure in children, as our sample included only 53 children, further research with larger cohort is required to validate these findings. Given the importance of the BDNF gene, it is unfortunate that our sample was missing subjects with the minor homozygote genotype for a full investigation of this gene’s effects. On the other hand, natural disasters provide unique opportunities to study the effect of an objective, randomly assigned stressor in a human population, thereby overcoming many limitations of previous human gene-by-environment research. The 1998 Quebec ice storm was a powerful enough stressor that high scores on the maternal objective hardship scale predicted a full standard deviation lower IQ in the children at age 2 (Laplante et al., 2004) and again at 5 1/2 years (Laplante et al., 2008), higher body mass index and obesity rates at ages 5 1/2 (Dancause et al., 2012) through age 15 (Liu et al., 2016), and higher insulin secretion (Dancause et al., 2013) and pro-inflammatory cytokines (Veru et al., 2015) at age 13. Consequently, using this population-level acute stressor, we have been able to delineate the influences of PNMS and SNPs on hippocampal volume in early adolescent offspring.

Our findings support the hypothesis that individuals exhibit differential susceptibility to the effects of PNMS, and that genetic variants that alter the function or expression of proteins, underlie these differences. We showed that, in some cases, genetic variants, PNMS, and the gene-by-environment interaction combined explained up to half the variance in hippocampal volume. This is significant as it increases our understanding of how genetic and environmental factors work in combination to affect hippocampal development.

## Conclusion

This work has increased our understanding of gene-by-environment interactions during prenatal brain development. Specifically, we have found that a SNP located on COMT significantly moderates the effects of PNMS on hippocampal volume, resulting in differential susceptibility between COMT genotypes to the effects of PNMS. In addition, the effect of different aspects of PNMS – objective hardship and subjective distress – on hippocampal volume was differentially moderated by the SNPs of interest. When moderated by SNPs located on COMT, subjective distress exhibited greater effects on hippocampal volume than objective hardship and cognitive appraisal in girls, while objective hardship had the most effect in boys. Overall, in accordance with our hypothesis, these results suggest that a child’s genotype can alter their vulnerability to the effects of PNMS; however, these effects are often specific to a particular sex and/or aspect of stress.

## Data Availability Statement

The raw data supporting the conclusions of this article will be made available by the authors, without undue reservation.

## Ethics Statement

The studies involving human participants were reviewed and approved by the Research Ethics Board of the Douglas Hospital Research Centre. Written informed consent to participate in this study was provided by the participants’ legal guardian/next of kin.

## Author Contributions

SK designed and implemented Project Ice Storm. LC-L, SY, and SK conceived of the current experiment. RD ran the automated segmentation pipelines, did the manual corrections of the amygdala, and total brain volume segmentations derived from the automated segmentations. GE, LC-L, and SY ran the statistical analyses. SY interpreted the data and drafted an early version of the manuscript. LC-L, SK, and DL provided intellectual contributions for the rationale, interpretation of the data, and prepared the final manuscript for submission. All authors contributed to the article and approved the submitted version.

## Conflict of Interest

The authors declare that the research was conducted in the absence of any commercial or financial relationships that could be construed as a potential conflict of interest.

## Publisher’s Note

All claims expressed in this article are solely those of the authors and do not necessarily represent those of their affiliated organizations, or those of the publisher, the editors and the reviewers. Any product that may be evaluated in this article, or claim that may be made by its manufacturer, is not guaranteed or endorsed by the publisher.

## Footnotes

1
info2.camh.net/kf-tigr/index.php/Hippocampus

2
cobralab.ca/software/MAGeTbrain
